# Bilio-entero-gastrostomy: prospective assessment of a modified biliary reconstruction with facilitated future endoscopic access

**DOI:** 10.1186/1471-2482-12-9

**Published:** 2012-06-21

**Authors:** Mostafa A Hamad, Hussein El-Amin

**Affiliations:** 1 Departments of General Surgery, Faculty of Medicine, Assiut University, Assiut, Egypt; 2 Departments of Medicine, Faculty of Medicine, Assiut University, Assiut, Egypt

## Abstract

**Background:**

Hepaticojejunostomy (HJ) is the classical reconstruction for benign biliary stricture. Endoscopic management of anastomotic complications after hepaticojejunostomy is extremely difficult. In this work we assess a modified biliary reconstruction in the form of bilio-entero-gastrostomy (BEG) regarding the feasibility of endoscopic access to HJ and management of its stenosis if encountered.

**Methods:**

From October 2008 till February 2011 all patients presented to the authors with benign biliary stricture who needed bilio-enteric shunt were considered. For each patient bilio-entero-gastrostomy (BEG) of either type I, II or III was constructed. In the fourth week postoperatively, endoscopy was performed to explore the possibility to access the biliary anastomosis and perform cholangiography.

**Results:**

BEG shunt was performed for seventeen patients, one of whom, with BEG type I, died due to myocardial infarction leaving sixteen patients with a diagnosis of postcholecystectomy biliary injury (9), inflammatory stricture with or without choledocholithiasis (5) and strictured biliary shunt (2). BEG shunts were either type I (3), type II (3) or type III (10). Endoscopic follow up revealed successful access to the anastomosis in 14 patients (87.5%), while the access failed in one type I and one type II BEG (12.5%). Mean time needed to access the anastomosis was 12.6 min (2-55 min). On a scale from 1–5, mean endoscopic difficulty score was 1.7. One patient (6.25%), with BEG type I, developed anastomotic stricture after 18 months that was successfully treated endoscopically by stenting. These preliminary results showed that, in relation to the other types, type III BEG demonstrated the tendency to be surgically simpler to perform, endoscopicall faster to access, easier and with no failure.

**Conclusions:**

BEG, which is a modified biliary reconstruction, facilitates endoscopic access of the biliary anastomosis, offers management option for its complications, and, therefore, could be considered for biliary reconstruction of benign stricture. BEG type III tend to be surgically simpler and endoscopically faster, easier and more successful than type I and II.

## Background

Benign biliary stricture is most commonly caused by operative injury of the common bile duct (CBD) during cholecystectomy or less frequently by inflammatory stenosis with or without stone formation, chronic pancreatitis, primary sclerosing cholangitis, strictured bilioenteric shunt and CBD injury due to penetrating trauma to the porta hepatis or during other operative procedures involving gastric, pancreatic, or hepatic resection [1-3]. Treatment of benign biliary stricture requires multidisciplinary approach including endoscopy, interventional radiology and surgery [4]. Whenever surgery is indicated, the procedure of choice is a Roux-en-Y hepaticojejunostomy (HJ) [3,5-8].

Stricture of the bilioenteric shunt following Roux-en-Y HJ is reported to range from 2% to 25% [2,3,7-10]. This is a major complication which is associated with repeated cholangitis and, if left untreated, with biliary cirrhosis and portal hypertension. Treatment could be by surgery, interventional radiology or endoscopy. Surgical treatment aiming at revision of the reconstruction of bilioenteric shunt is often difficult and represents major challenge to the surgeon [11]. Percutaneous transhepatic interventional therapy could be effective but is invasive especially to the liver which is often the seat of impaired function or overt cirrhosis [12,13].

Endoscopic management is not only the least invasive but also very effective via either balloon dilatation or stenting of the stricture. The main obstacle for endoscopic approach is the extremely difficult access of the endoscope to the bilioenteric shunt due to the altered anatomy of the Roux-en-Y construction. Some authors advocated the use of double [14] or single [15] balloon enterostomy to overcome this difficulty. Still, however, the procedure is difficult and time consuming. Others construct surgical access loop to be used by the endoscopist to reach the HJ stoma. These access loops could be jejunocutaneous either superficial [16] or subfacial [17], jejunal loop interposition [18], jejunoduodenostomy [19] or gastric access loop [20-22].

This study is a consecutive case series in which we prospectively assess bilio-entero-gastrostomy (BEG), which is a modified bilioenteric shunt with gastric access loop, regarding the feasibility of endoscopic access to HJ and management of its stricture if encountered. Additionally, we compare three different construction techniques of BEG in this regard.

## Methods

This is a prospective study, in the form of consecutive case series, the protocol of which was approved by the ethical committee of Assiut Faculty of Medicine. Included in the study are all patients presenting to the authors with a diagnosis of benign biliary stricture for which surgical correction is indicated in the period from October 2008 till February 2011. For those patients other therapeutic options as percutaneous transhepatic cholangiography (PTC) or endoscopic retrograde cholangiopancreatography (ERCP) dilatation or stenting were either not applicable or not successful.

For all patients, full medical history, clinical examination, laboratory investigations in the form of liver function tests (LFT), prothrombin time and concentration (PTT), complete blood count (CBC) and kidney function tests (KFT) were performed. Imaging studies were also carried out in the form of abdominal ultrasonography (US), computerized tomography scan (CT) of the abdomen and magnetic resonance cholangiography (MRC) if indicated. ERCP and/or PTC were performed, whenever applicable, whether for diagnosis or therapeutic trial. Thorough preoperative medical fitness assessment was done before surgery.

**Figure 1:**
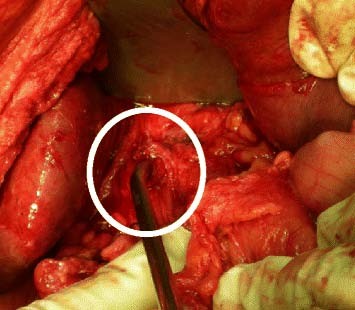
Preparation of the proximal CBD stoma.

**Figure 2:**
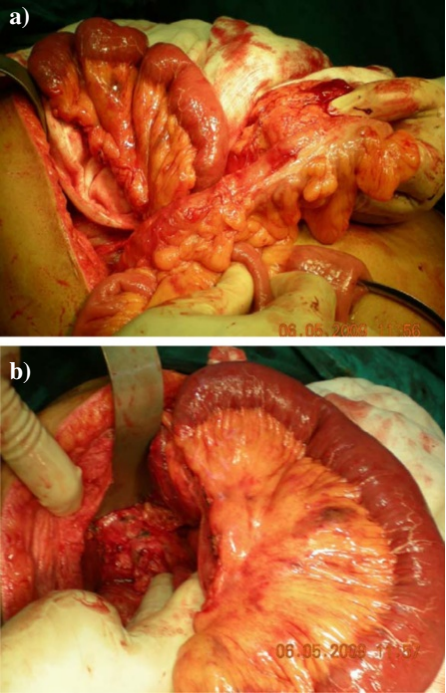
a) Preparation of the Roux jejunal loop after lengthening of the mesentery. b) Passing the loop retrocolic.

**Figure 3:**
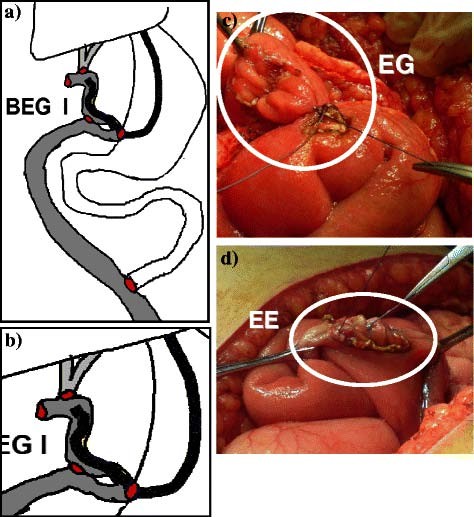
**BEG type I.a**) and **b**) Diagrams of the construction.** c**) Enterogastrostomy [EG]. **d**) Enteroenterostomy [EE].

**Figure 4:**
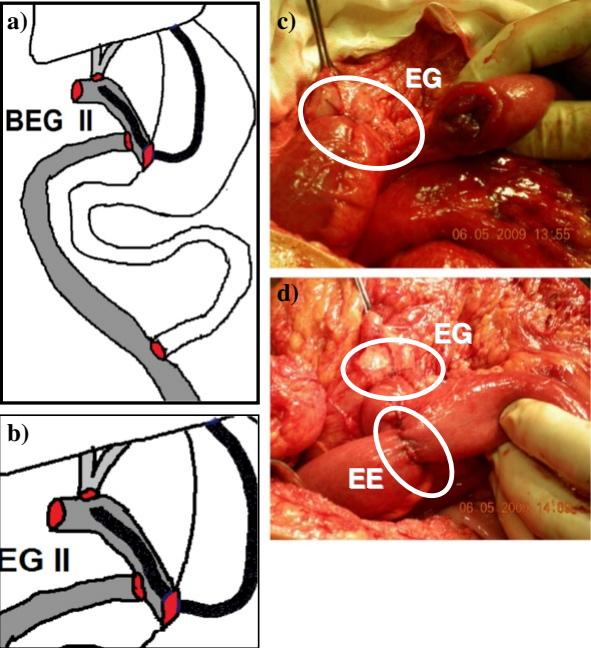
**BEG type II.a**) and **b**) Diagrams of the construction. **c**) Enterogastrostomy [EG]. **d**) Enteroenterostomy [EE].

**Figure 5:**
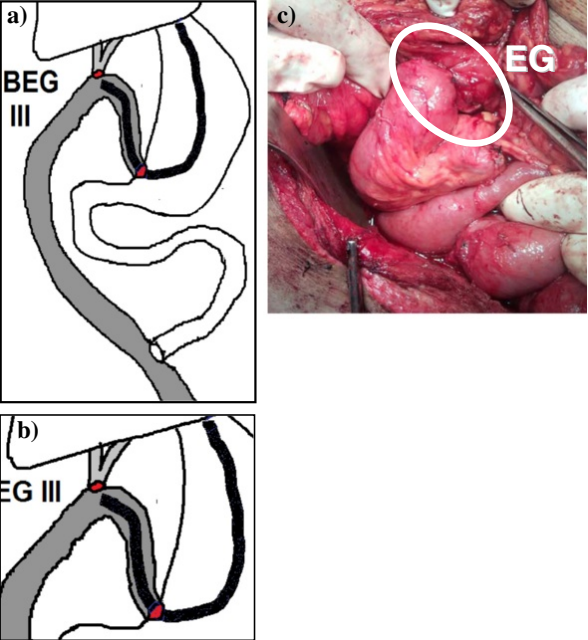
**BEG type III.a**) and **b**) Diagrams of the construction. **c**) Enterogastrostomy [EG].

Surgical technique: under general intubation anesthesia after administration of prophylactic antibiotic in the form of third generation cephalosporin, a generous right subcostal incision was performed and could be extended on demand upward to the xyphoid process and/or to the left subcostal area. Thorough dissection and adesiolysis was performed to reach the CBD and prepare the unaffected proximal part for anastomosis (Figure 1). The Roux jejunal loop was prepared with lengthening of the mesentery and then passed retrocolic to reach the porta hepatis (Figure 2). Afterwards, BEG was constructed depending on the type; I, II or III. In all types, the HJ is end to side anastomosis using interrupted sutures of polyglactin 910 of 4–0 size. The level of HJ is dependent on Bismuth classification [10] of the level of the stricture and also on the diameter of CBD and the operative circumstances. If the level was at the carena (Bismuth type III), the HJ could be performed, using the Hepp-Couinaud technique [23], with widening of the stoma by extension through the wall of the left hepatic duct. When the two ducts constituted separate openings (Bismuth type IV), the septum in between, if applicable, could be sutured and cut to transform both ducts into single stoma. The rest of the anastomoses, including the enterogastrostomy (EG), are constructed according to the type of BEG. All JG are anastomosed to the anterior wall of the stomach as near as possible to the pyloric orifice. In BEG type I (Figure 3), a side loop of the Roux jejunum is anastomosed at its apex to the stomach with another side to side enteroenterostomy as a conduit of bile away from the stomach. In BEG type II (Figure 4), the Roux jejunum is transected about 10 to 15 cm from HJ without interference with its mesentery. The distal end is anastomosed to the stomach, while the proximal end is anastomosed to the side of the distal loop as a conduit of bile. In BEG type III (Figure 5), HJ is constructed leaving the distal end of the Roux jejunum long enough to be anastomosed to the side of the stomach. All the enterogastrostomies and enteroenterostomies were in the form of single-layer continuous sutures of polyglactin 910 of 3–0 size. An intraperitoneal drain was left in the hepatorenal pouch before closing the incision.

The choice of the type of BEG was an evolving process. We began with the BEG type I. The endoscopist faced difficulty with this technique due to the presence of more than one lumen at the site of gastroenterostomy making the decision and technique of entery more complex. Consequently, we modified the reconstruction to the BEG type II which appeared to be relatively easier for the endoscopist. However, some difficulty was still present for the endoscopic approach due to the side to end enteroenterostomy needed for this type. Moreover, BEG type II was more complex to be performed surgically. Consequently the technique was further modified to BEG type III which was not only the simplest to perform surgically but also the easiest for the endoscopist to reach the anastomosis due to the presence of only one lumen at the site of the enterogastrostomy. This may explain the sequence of cases in relation to the type of BEG, the increased number of BEG type III and the concentration of the cases with this type toward the later period of the study. Consequently, toward the end of the study, we decided to perform all cases using BEG type III.

Postoperatively, antibiotics in the form of third generation cephalosporin were administered twice daily for at least five days. The patients were closely followed up for any bile leak, improvement in the LFT, restoration of gastrointestinal motility and oral feeding. The patients were discharged from the hospitals once they were well mobilized, oral feeding was restored, and LFT were relatively improved.

Follow up visits, after hospital discharge, were scheduled weekly for the first four weeks then monthly for three months then every six months or whenever there were abnormal symptoms. In each visit, the patients underwent clinical assessment specially manifestation of cholangitis and laboratory assessment specially serum bilirubin and alkaline phosphatase levels. In the fourth postoperative week, endoscopic assessment of the BEG shunt with evaluation of the HJ was scheduled for all patients.

Endoscopic technique: within the 4^th^ postoperative week, gastroenteroscopy was performed using end-view gastroduodenoscope (pentax 3440, Tokyo, Japan). The aim was to assess the feasibility to access the HJ stoma and perform cholangiography. With the patient in the left lateral position, monitored anesthesia care (MAC) sedation with propofol was used with an initiation dose of 100–150 mcg/kg/min for a period of 3–5 minutes and a maintenance dose of 25–75 mcg/kg/min which was adjusted to clinical response. The endoscope was introduced through the esophagus to the stomach where we assessed the amount of bile in the stomach on a scale of 0 to 2 where 0 meant no bile, 1 meant minimal amount of bile staining the gastric mucosa, and 2 meant large amount of bile accumulating and needed to be sucked. The gastroenterostomy stoma was assessed as regard to its site, diameter, as well as the difficulties we faced to pass through it. We passed through the gastroenterostomy either directly with the scope or over a guiding catheter. Thereafter, when we reached the HJ stoma, we inject a diluted dye into the cannulated bile ducts using an ERCP catheter to obtain cholangiography. Time to reach the gastroenterostomy as well as time to reach the HJ was reported for each case. Failure of endoscopic access was defined as failure to reach the HJ and perform cholangiography. For failed cases, the cause of failure and the type of BEG performed were reported. After the end of each procedure, the endoscopist was asked to score the difficulty of it on a scale from 1 to 5 where 1 is the easiest and 5 is the most difficult. It is worth mentioning that all endoscopies were performed with the same endoscopist (HE) who has more than fifteen years of experience in gastrointestinal endoscopy including ERCP and therapeutic endoscopies.

During the follow up, if the patient suffered cholangitis that required medical treatment, imaging studies were performed including abdominal US and MRC to exclude the presence of HJ stricture. If stricture did exist, endoscopic diagnosis and therapeutic trial was performed aiming at either balloon dilatation and/or stent placement through the strictured anastomosis. According to Terblanche grading of clinical outcome of HJ (Table 1) [24], the patients were assessed and categorized.

**Table 1:** Terblanche clinical grading of long term clinical results of hepaticojejunostomy

**Grade I**	**Excellent**	**No biliary symptoms**
Grade II	Good	Transitory symptoms / No treatment
Grade III	Fair	Biliary symptoms requiring medical therapy
Grade IV	Poor	Biliary symptoms due to recurrent stricture requiring intervention

All data were prospectively collected for assessment and for comparing the results of the three different types of BEG.

## Results

In the period of the study, seventeen cases of benign biliary stricture were treated by the authors using BEG shunt. One patient, with BEG type one reconstruction, died of myocardial infarction on the second postoperative day and was excluded from the study. This left sixteen patients to be included. Seven patients were males and nine were females. The age ranged from 22 to 72 years with a mean age of 40.9 years.

**Figure 6:**
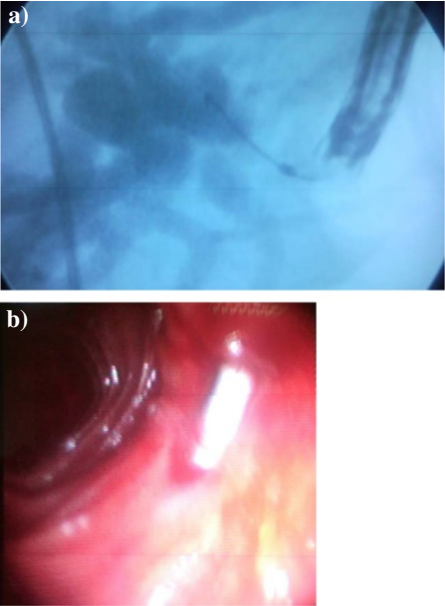
**Endoscopic stenting of the case complicated by anastomotic stricture.a**) Fluroscopic view of the stent pushed in place over the guide wire. **b**) endoscopic view of the stent in place.

Tables 2, 3 and 4 show the main preoperative, operative, and postoperative data of the patients respectively. The diameter of the proximal biliary channel above the level of the stricture ranged from 6 mm to 20 mm. One patient (6.26%) with BEG type I developed HJ stricture about 18 months after the operation. The patient presented with recurrent cholangitis and MRC revealed strictured HJ. Endoscopic cholangiography was performed and the stricture was dilated with placement of a ten-french stent through it (Figure 6). The patient was followed up for eleven months with uneventful course.

**Table 2:** Preoperative data of the patients

**Data**	**Description**		**No. (%)**		**Total**
Diagnosis	Post-cholecystectomy CBD injury	Immediate leak	3	9 (56.25%)	16
		Immediate obstruction	3		
		Late Stricture	3		
	Strictured Hepatico-Jejunostomy (after original post-cholecystectomy CBD injury)		2 (12.50%)		
	Inflammatory stricture (cholangitis)	With CBD stones	3	5 (31.25%)	
		Without CBD stones	2		
Bismuth type of stricture	I (>2 cm from the confluence)		5 (31.25%)		16
	III (<2 cm from the confluence)		3 (18.75%)		
	IIII (hilar with preserved confluence)		7 (43.75%)		
	IV (hilar with separate right and left ducts)		1 (6.25%)		
	VI (sectorial right hepatic duct)		0 (0%)		
Liver functions	Total bilirubin (μmol/L)	Elevated	14(87.50%)		16
		Normal	2 (12.50%)		
	Alkaline phosphatase (U/L)	Elevated	16 (100%)		16
		Normal	0 (0.0%)		
Cholangio-graphy	ERCP	Number of procedures	21		16*
		Number of patients	14 (87.50%)		
	MRC	Number of procedures	7		
		Number of patients	7 (43.75%)		

CBD: common bile duct, ERCP: endoscopic retrograde cholangiopancreatography, MRC: magnetic resonance cholangiography, * represents the total number of patients that had cholangiography whatever the type.

**Table 3:** Operative data of the patients

**Data**	**Description**	**No. (%)**	**Total**
Level of bilioenteric shunt from the carena	≥2 cm	5 (31.25%)	16
	<2 cm	3 (18.75%)	
	At the carena (classic technique)	2 (12.50)	
	At the carena (left duct extension-Hepp Couinaud technique)	5 (31.25%)	
	Two separate ducts (septum was sutured and cut to form one stoma)	1 (6.25%)	
Type of BEG	Type I	3 (18.75%)	16
	Type II	3 (18.75%)	
	Type III	10 (62.50%)	
Other procedures	Yes (cholecystectomy)	5 (31.25%)	16
	No	11 (68.75%)	
Operative time (min) Mean (range)	227.5 min (150–350 min)		

BEG: bilioenterogastrostomy.

**Table 4:** Postoperative data of the patients

**Data**	**Deascription**	**No. (%)**	**Total**
Clinical improvement	1 week	10 (62.50%)	16
	2 weeks	6 (37.50%)	
	≥3 weeks	0 (0%)	
Laboratory improvement	1 week	3 (18.75%0	16
	2 weeks	7 (43.75%)	
	≥3 weeks	6 (37.50%)	
Hospital stay Mean (range) days			
Long term clinical results (cholangitis)	Grade I (excellent)	12 (75.00%)	16
	Grade II (good)	2 (12.50%)	
	Grade III (fair)	1 (6.25%)	
	Grade IV (poor)	1 (6.25%)	
Follow up period Mean (range) months			
HJ stricture	One case, presented after 18 months	1 (6.25%)	16
	BEG type I, endoscopic stent,		
	Normal after 9 months follow up		
	Patent HJ	15 (93.75%)	

HJ: hepaticojejunostomy.

Endoscopic data are shown in Table 5. Endoscopic access to the HJ was successful in 14 cases (87.5%) while failed in 2 cases (12.5%). The first failure was in case number two with BEG type I. The enterogastrostomy (EG) was impossible to be entered by the endoscope as it was placed high on the lesser curve of the stomach. Afterwards, all EG anastomoses were placed as near to the pyloric orifice as possible. The second failure was in case number seven with BEG type II. The EG was too narrow to pass the endoscope, however the endoscopist stated that whenever the access is mandatory, the EG could be dilated enough to accommodate the endoscope. In spite of that, we considered this case as a failure to endoscopic access of the HJ.

**Table 5:** Endoscopic data of the patients

**No**	**BEG type**	Time to reach JG*	**Access to HJ**	Time to reach HJ*	**Difficulty score (1–5)**	**Bile in stomach score (0–1)**
1	I	3	Succeeded	24	3	1
2	I	2	Failed	Failed	Failed	1
3	II	2	Succeeded	10	2	2
4	I	2	Succeeded	55	4	1
5	II	2	Succeeded	20	3	1
6	III	1	Succeeded	7	1	0
7	II	2	Failed	Failed	Failed	1
8	III	2	Succeeded	12	2	1
9	III	2	Succeeded	10	1	2
10	III	1	Succeeded	8	1	2
11	III	1	Succeeded	12	2	0
12	III	1	Succeeded	2	1	0
13	III	2	Succeeded	7	1	1
14	III	2	Succeeded	6	1	1
15	III	1	Succeeded	2	1	1
16	III	1	Succeeded	2	1	2
Mean		1.6		12.6	1.7	1

*Time is in minutes.

Table 6 compares between the three types of BEG. It is shown that type III BEG tend to be better than the other two types regarding the success rate of access to HJ, time to reach HJ, and difficulty to the endoscopist. Additionally, BEG type III is easier to perform requiring only three anastomoses per case while the other two types require five anastomoses. However, further assessment may be needed due to the small number of cases and the difference in number between the groups.

**Table 6:** Comparison between the three types of BEG

**Type**	**No. of cases**	**No. of Anast. per case**	**Success of Endoscopic access to HJ**	**Time to reach HJ (min)**	**Difficulty score (mean score)**	**Bile in Stomach (mean score)**
BEG I	3	5	2 (66%)	39.5	3.5	1
BEG II	3	5	2 (66%)	15	2.5	1.3
BEG III	10	3	10 (100%)	6.8	1.2	1

BEG: bilioenterogastrostomy, HJ: hepaticojejunostomy, Anast.: anastomoses.

## Discussion

Roux-en-Y hepaticojejunostomy is the most commonly used surgical technique for reconstruction of the biliary tract in patients with benign biliary stricture [3,5-8]. In those patients, there is a relatively high incidence of strictured HJ ranging from 2% to 25% [2,3,7-10]. The endoscopic management of these complications seems to be the best option and least invasive compared to interventional radiology or reoperation [11-13]. However, the altered anatomy of the Roux-en-Y construction represents a major difficulty to access the HJ by the endoscope. The use of standard gastroduodenoscope proved to be extremely difficult, time consuming and with high failure rate [14,15].

Several attempts were reported to overcome these difficulties in endoscopic approach to HJ. The use of long enteroscope, whether double balloon or single balloon, was shown to improve the results of endoscopic access. However, these reports are still few with limited number of cases and the availability of the enteroscopes and their assigned accessories are still not universally present in all endoscopy units [14,15].

On the other hand, several modifications of the surgical technique of the HJ have been suggested to enable access to the shunt either by interventional radiology or endoscopy. Some authors recommended an associated jejonostomy to be used for access for one year postoperatively and closed afterwards [16]. However, other authors tried to overcome the inconvenience of the stoma to the patient in addition to the possibility of delayed shunt problems later than the suggested one year period by making perminant jejunostomy subcutaneously and open it under local anesthesia whenever the access is needed then close it again [17]. Another approach is to construct an interpositioned jejunal loop to act as a conduit between the CBD and the duodenum [18]. It was found that a jejunal loop less than 20 cm in length will help the endoscope pass through but a loop more than 40 cm will prevent recurrent ascending cholangitis; a controversy that is difficult to solve [18,25]. Other authors recommended the use of duodenal access through side jejunoduodenostomy [19] or gastric access through enterogastrostomy [20-22].

To our knowledge, there are three series of gastric access loop in the literature that will be discussed in view of the available data. Sitaram et al. [20] reported the first series in ten patients. They succeeded to enter the enterogastrostomy in five patients only. In another study, Selvakumar et al. [21] reported eleven patients with gastric access loop with 73% success rate of endoscopic access of the HJ stoma. The failed three cases were due to strictured enterogastrostomy. Their follow up period was relatively long (mean 51 months). They encountered one strictured HJ (9%) which did not benefit from the access loop. The patients did not have clinical or endoscopic evidence of bile gastritis. In the third study, Jayasundara et al. [22] reported 27 patients who had bilioenteric shunt with gastric access loop. Three patients (11%) developed strictured HJ that were successfully managed endoscopically through the access loop. During the follow up period of about two and half years, they did not discover gastric access related morbidity using the dyspepsia disability score. In our series with 16 cases, we assessed different construction of gastric access loop in the form of bilioenterogastrostomy (BEG) type I, II and III regarding endoscopic access of the HJ. We found that the overall success rate of endoscopic access to the HJ through the three types of BEG was 87.5%, while it was 100% for BEG type III, which is a construction similar to the three series that were discussed previously [20-22]. These success rates compared favorably with other similar series [20,21] except Jayasundara et al. [22]. In spite of having bile in the stomach in most of the cases, the patients did not show any clinical manifestations of biliary gastritis which is similar to the results of other authors [20-22]. The endoscopist considered the procedure easy with a mean score of 1.7 and 1.2 for the whole series and for BEG type III respectively on a scale from 1 to 5. The mean procedure time was convenient reaching 12.6 minutes for the whole series and 6.8 minutes for BEG type III. Our cases were a mixture of different indications for BEG including two difficult cases of revision of strictured HJ. Also, different levels of HJ were reported from Bismuth type I to type IV. We think that these wide varieties constitute a good assessment of the BEG technique. We had a follow up period with a mean of 23 months that was comparable to the other similar series [20,22] except for Selvakumar et al. [21] who reported a mean of 51 months follow up. We think that our follow up period would better be extended to have long term results that can better reflect the real comlication rate. We had one strictured HJ (6.25%) which compared favorably with the other series [20-22]. This case was successfully treated endoscopically by stenting which proved the efficacy of BEG construction in facilitating endoscopic management of complicated bilioenteric shunt as was previously shown by other authors [22].

Regarding the three types of BEG, type I and II were more complex in construction with five anastomoses per case and meant to prevent bile reflux in the stomach in relation to type III. These two types, however, did not improve the bile reflux. Additionally, we found that BEG type III, which was easier to perform with only three anastomoses per case, was more successful in accessing HJ, required shorter time to reach it, was easier for the endoscopist. This explains why, from the middle of the series, we preferred to use BEG type III and abandoned the other two types.

## Conclusions

In conclusion, bilioenterogastrostomy (BEG), which is a modified biliary reconstruction using a gastric access loop, facilitates endoscopic access of the biliary anastomosis, offers management option for its future complications, and, therefore, could be considered as an advantageous option for reconstruction of benign biliary stricture. BEG type III tend to be surgically simpler and endoscopically faster, easier and more successful than type I and II.

## Competing interests

The authors declare that they have no competing interests.

## Authors’ contributions

MAH conceived the idea and design the operative protocol. He was also the main operator for all cases. In addition, the participated in drafting the manuscript. HE designed the endoscopic protocol, was the endoscopist of all cases, and revised the manuscript. All authors read and approved the final manuscript.

## Pre-publication history

The pre-publication history for this paper can be accessed here:

http://www.biomedcentral.com/1471-2482/12/9/prepub
